# Clinical and Magnetic Resonance Imaging Findings of Neurotoxocariasis

**DOI:** 10.3389/fneur.2018.00053

**Published:** 2018-02-08

**Authors:** Sofia S. Sánchez, Hector H. García, Alessandra Nicoletti

**Affiliations:** ^1^Department of Microbiology, School of Public Health (SSS), Center for Global Health – Tumbes (HHG), Universidad Peruana Cayetano Heredia, Lima, Peru; ^2^School of Sciences (HHG), Center for Global Health – Tumbes (HHG), Universidad Peruana Cayetano Heredia, Lima, Peru; ^3^Cysticercosis Unit, Instituto Nacional de Ciencias Neurologicas, Lima, Peru; ^4^Department G. F. Ingrassia, Section of Neurosciences, University of Catania, Catania, Italy

**Keywords:** toxocariasis, central nervous system infections, neurotoxocariasis, helminths, myelitis

## Abstract

Human toxocariasis is one of the most prevalent helminthiases worldwide. *Toxocara canis* larvae can cross the blood–brain barrier leading to the neurotoxocariasis. The clinical presentation consists of a wide spectrum of neurological manifestations, but asymptomatic infection is probably common. Neurotoxocariasis is not a frequent diagnosis probably due to the non-specific nature of its symptoms as well as the lack of confirmatory diagnostic tests. Diagnosis of neurotoxocariasis is based on the presence of a high titer of anti-*Toxocara* antibody in the cerebrospinal fluid or in the serum, presence of eosinophilia in the serum or cerebrospinal fluid, and clinical and radiological improvement after anthelmintic therapy; however, universally accepted diagnostic criteria are lacking. Magnetic resonance imaging (MRI) findings include single or multiple, subcortical, cortical or white matter hyperintense lesions, best visualized on FLAIR and T2-weighted imaging, and usually isointense or hypointense on T1. These imaging findings are suggestive but not specific to neurotoxocariasis. Definitive diagnosis is made by histological confirmation, but it is rarely followed. This review provides an overview of the clinical manifestations, management options, and MRI findings of neurotoxocariasis.

## Introduction

### Life Cycle and Epidemiology

Human toxocariasis is a parasitic zoonosis caused by the larval stages of the ascarids *Toxocara canis*, the common roundworm of dogs, and probably by the roundworm of cats (*Toxocara cati*) as well. Among the helminthiases, toxocariasis is one of the most prevalent worldwide, especially in settings where human–soil–dog contact is particularly common. Even if the parasite tends to be more prevalent in tropical settings, seroprevalence reaches up to 80–90% in Western countries ranging from 35 to 42% in rural areas and from 2 to 5% in urban areas (1).

*Toxocara* is a nematode that usually inhabits the small intestine of the host; dogs can become infected by transplacental spread or by contact with contaminated feces. The female *Toxocara* produces up to 200,000 eggs per day, releasing them to the environment through the dog’s feces (2). Humans can become infected by direct contact with dogs or by the ingestion of contaminated food or soil. Ingested eggs develop into juvenile larvae that cross the small intestine and migrate to any organ through the circulatory system, resulting in a multisystemic inflammatory tissue reaction. Visceral larva migrans (VLM) and ocular larva migrans are the most common clinical manifestations (3). More recently, two different syndromes have been described: covert toxocariasis, which is more common in children and common toxocariasis, reported in adults, both characterized by mild *Toxocara* infection and less-severe systemic manifestations (4).

### Central Nervous System (CNS) Infection

*Toxocara canis* larvae can cross the blood–brain barrier, invading the CNS, leading to neurotoxocariasis. Autopsy studies have revealed *T. canis* larvae in the leptomeninges, both the gray and white matter of the cerebrum, the cerebellum, and the spinal cord (5–10). However, most of these cases did not have clinical neurological signs and, for this reason, the clinical significance and true frequency of cerebral localization of *Toxocara* larvae remain unknown. To date, CNS infestation of *T. canis* larvae in humans is thought to be rare, even if in animal models larvae often migrate to the brain (11).

Asymptomatic CNS infection is probably common, clinically apparent neurotoxocariasis consists of a wide spectrum of neurological manifestations from meningitis, encephalitis, and myelitis, to cerebral vasculitis (1). Peripheral nervous system manifestations of toxocariasis have been reported in a few cases and comprise radiculitis, affection of cranial nerves, or musculo-skeletal involvement (12). According to two recent reviews, approximately 100 cases of neurotoxocariasis have been reported in the literature since 1956. A possible association between *T. canis* and epilepsy has been suggested by several case–control studies (13, 14).

### Diagnosis of Neurotoxocariasis

Neurotoxocariasis is not a frequent diagnosis and it is probably underdiagnosed due to the non-specific nature of its symptoms (seizures and headache) exacerbated by a lack of confirmatory diagnostic tests. Diagnosis of neurotoxocariasis is based on the presence of a high titer of anti-*Toxocara* antibody in the CSF or in the serum, presence of eosinophilia in the serum or CSF, and clinical and radiological improvement after anthelmintic therapy (12, 15), but validated diagnostic criteria are lacking. The standard serological test is an ELISA based on secretory-excretory antigens (TES) from *Toxocara canis* larvae of the second stage (16). These antigens are a mixture of several glycoproteins that have Th2 type immune response with high levels of interleukin (IL) 4 and IL5. ELISA for the detection of specific IgG antibodies to TES in serum has a sensitivity of 78% and a specificity of 92% for the diagnosis of VLM, although cross-reactions with other nematode infections reduce its specificity, particularly in tropical areas (17, 18). As for any antibody test, a positive IgG ELISA cannot distinguish between past and current infection, although decreasing antibody titers after antihelminthic treatment or following clinical remission are highly suggestive of the diagnosis (18).The use of fractionated native TES in the western blot (WB) assay overcomes the issues with cross-reactions to other nematodes in ELISA assays because the low molecular weight bands (24–32 kDa) are specific for *Toxocara* infection (19). Unfortunately, western blotting is more expensive and labor-intensive than ELISA, and thus screening with the indirect TES–IgG–ELISA, followed by confirmation with the TES–WB, is considered an effective approach (18, 20).

A variety of brain imaging findings have been reported in relation to neurotoxocariasis, initially using computed tomography and more recently, using magnetic resonance imaging (MRI). Despite the evident importance of characterization of neuroimaging findings, few studies have evaluated neurotoxocariasis by MRI. We carried out a systematic review to investigate the clinical manifestations, management options, and MRI characteristics of cerebral toxocariasis.

## Methods

We used the following search strategy (((“toxocariasis”) OR (“toxocariosis”) AND “central nervous system”) OR “neurotoxocariasis”) OR (“*Toxocara canis*” AND “central nervous system”) to identify neurotoxocariasis cases recorded in PubMed until March 1, 2017. We evaluated articles in English, Spanish, Portuguese, and French that reported original data of case reports or case series. We included only cases that underwent an MRI study. Cases describing peripheral nervous system involvement were not included because the main aim of this review was to evaluate the MRI characteristics of neurotoxocariasis in the CNS. Epidemiological studies such as case–control studies assessing the association between neurotoxocariasis and epilepsy were also excluded. A search of the reference lists of the identified articles led to identification of further relevant articles.

## Clinical and MRI Findings

Since the first reported case of neurotoxocariasis who underwent a brain MRI, published in 1991, we identified 48 articles (21–68) including three case series (25, 27, 28) involving a total of 104 patients with neurological manifestations in the CNS [either myelitis (*n* = 70, 67.3%) or cerebral toxocariasis (*n* = 34, 32.7%)]. Most patients were men (79, 76.0%) and their mean age was 42.3 ± 15.2 years (SD). Histological confirmation was obtained in only seven cases. Detailed information on the 104 reported cases is provided in Table S1 in Supplementary Material.

### Myelitis

Isolated myelitis represents the most commonly reported presentation, occurring in 70 cases [61 men (87.1%); mean age 45.1 ± 9.8 years] (21–36); three of them were classified as meningomyelitis due to the presence of local leptomeningeal involvement (30, 31, 34).

The majority of cases were reported in three different case series including 17, 8, and 31 cases (25, 27, 28). Clinical manifestations included sensory and motor disturbances, predominantly involving the lower limbs and often accompanied by autonomic dysfunction. Liver or lung involvement was reported in nine patients (12.9%) (26, 28).

**Figure 1:**
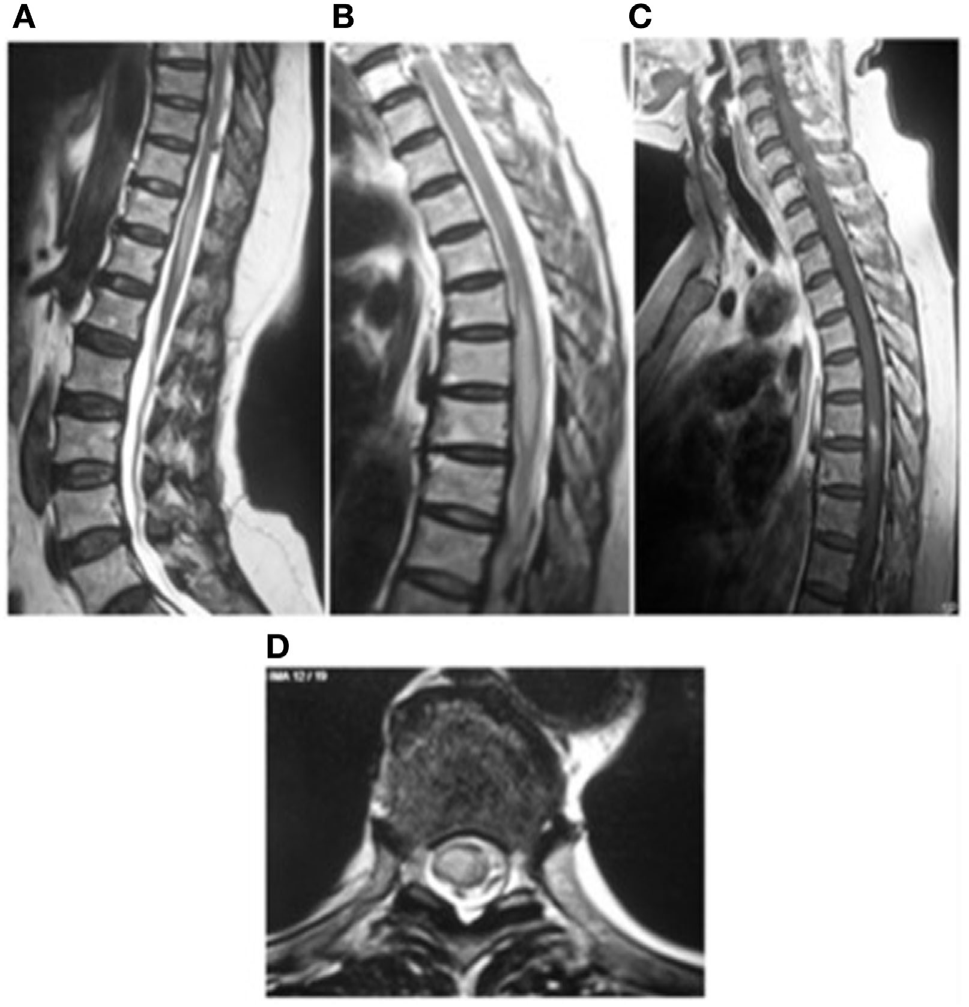
Myelitis (21) **(A,B)** sagittal T2-weighted image of spine showing high signal intensity from T5 to T7 and T10 to T12 levels. **(C)** T1-weighted images with contrast enhancement. **(D)** High signal intensity in the spinal cord in the sagittal images. This image has permissions for use.

**Figure 2:**
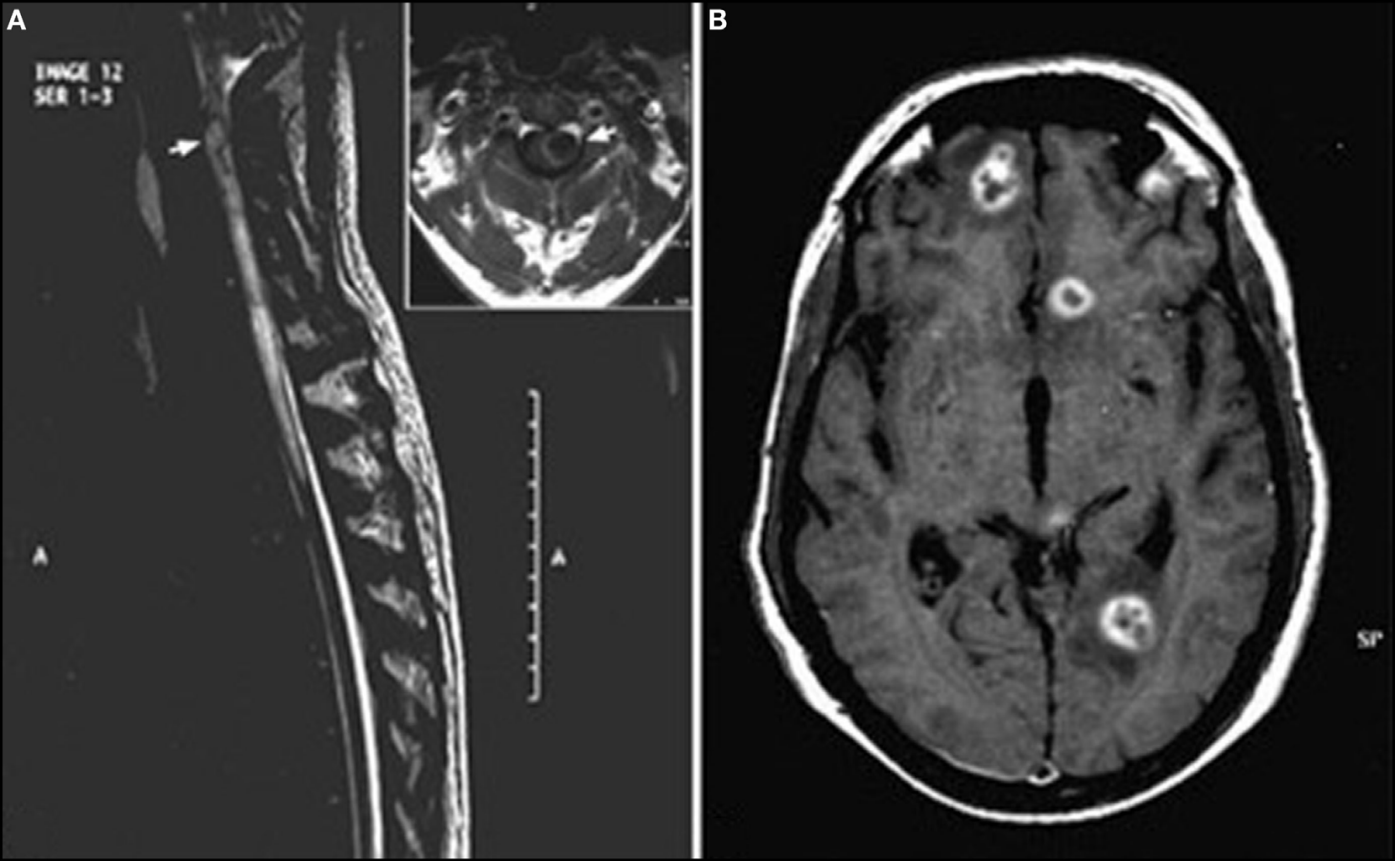
Encephalomyelitis (50) **(A)** T2-weighted sagittal image of spine showing a hyperintense lesion (arrow) with extensive perilesional edema in the upper cervical cord. Inset highlights large lesion (arrow) with peripheral hyperintense rim and perilesional edema causing expansion of cervical cord. **(B)** T1-weighted axial post-contrast image of brain shows multiple ring-enhancing lesions in bilateral frontoparietal deep white matter with nodular enhancement of the wall seen in left frontal lesion. This image has permissions for use.

On MRI, spinal toxocariasis is characterized by swelling and enlargement of the involved spinal segment and hyperintensity on T2 and FLAIR sequences with focal nodular enhancement after gadolinium injection (Figure 1). The enhancing area is generally smaller than the affected area with a tendency to involve the posterior-lateral areas of the cord (Figure 2). Lesions varied in size; they were almost always singular and located at the thoracic and cervical levels (Table S1 in Supplementary Material).

ELISA for anti-*Toxocara* antibodies in the CSF was assessed in 38 patients and was positive in all (100%); a confirmatory WB in CSF was available for 20 patients. For 31 patients (44.3%), the diagnosis was based on ELISA seropositivity, confirmed by WB only in 9 patients (24 presented also elevated serum IgE); in 1 patient with serum antibodies detected by ELISA, the diagnosis was confirmed by biopsy (23).

Mild–moderate blood eosinophilia was reported in 36 cases (51.4%) while CSF analysis was performed in 62 cases of whom only 21 (33.9%) presented with CSF eosinophilic pleocytosis (Table S1 in Supplementary Material; Table 1). When reported (39 cases) IgE level was always high (>150 U/ml). Diagnosis was confirmed by biopsy in only one of the patients with eosinophilia (23).

**Table 1:** Blood and CSF characteristics.

	Myelitis (*n* = 70)	Cerebral toxocariasis (*n* = 34)
Peripheral eosinophilia	36/70 (51.4%)	28/33 (84.9%)
CSF pleocytosis	21/62 (33.9%)	19/29 (65.5%)
Positive serum ELISA	69^a^/70 (98.6%)	32/32^d^ (100%)
Positive serum western blot (WB)	29/32 (90.6%)	8/8 (100%)
Positive CSF ELISA	38/38 (100%)	18^b^/23 (78.3%)
Positive CSF WB	20/20 (100%)	8^c^/8 (100%)
Biopsy	1/70 (1.4%)	6/34 (17.6%)

*^a^Two cases = enzyme immunoassay (EIA)*.

*^b^One case = EIA*.

*^c^One case = CSF PCR*.

*^d^One case = immunofluorescence*.

Fifty-four patients (77.1%) received anthelmintic treatment (albendazole in 50 cases), almost always with concomitant corticosteroid therapy (*n* = 52), while corticosteroids alone were administered only in 16 cases. After treatment, almost all patients improved clinically (only one remained stable) and radiologically, including those treated only with corticosteroids.

Changes in antibody titers after treatment were only evaluated in a few cases and at different follow-up times with conflicting results (24, 28, 31, 33, 34).

### Cerebral Toxocariasis

Encephalic localization has been reported in 34 patients [18 men (54.5%); mean age 33.5 ± 21.3 years]; 2 patients were classified as meningitis (38, 52), 1 as encephalitis (37), 4 as meningoencephalitis (46, 53, 54, 58), 3 as encephalomyelitis (29, 49, 51), 1 as meningoencephalomyelitis(55), 2 as cerebral abscess (41, 50), and 1 as obstructive hydrocephalus (61). Thirteen were generically defined as cerebral toxocariasis (21, 39, 40, 42–45, 47, 48, 56, 57, 59, 60) and seven were classified as vasculitis (62–68).

Encephalic/meningeal toxocariasis involvement was associated with a wide range of clinical manifestations including headache, seizures, focal deficits, confusional state, and cognitive impairment, with or without fever. A case of obstructive hydrocephalus due to toxocariasis was reported in a 46-year-old man who developed headache, diplopia, and visual deficits (61). Concomitant liver or lung involvement was reported only in two patients. CNS vasculitis was a rare and severe presentation that manifested as headache, cognitive impairment, and acute ischemic events and was described in seven patients; in three of them, liver or lung involvement was also reported (62–64).

Magnetic resonance imaging findings are not specific, showing single or multiple subcortical, cortical, or white matter hyperintense lesions on FLAIR and T2WI, usually isointense or hypointense in T1WI (Figure 1). Homogeneous or punctate enhancement after gadolinium injection was observed in nine cases, and single or multiple ring-enhancing lesions were described in four cases (Figure 2) (41, 42, 44, 50). Focal meningeal contrast enhancement, next to an active inflammatory lesion, was described in three patients (55, 59, 61). In the patient with obstructive hydrocephalus (61), brain MRI revealed hydrocephalus and leptomeningeal enhancement at the prepontine cistern, left cerebellopontine angle cistern, bilateral cerebral hemisphere, and a non-enhancing mass posterior to cerebral aqueduct. Imaging of the patients with vasculitis generally showed ischemic lesions; in four of the seven cases, diffusion-weighted imaging sequences were also performed demonstrating the presence of multifocal acute infarctions (62, 63, 66, 67). MRI angiography was performed only in one case (65) showing numerous segmental and irregular stenoses of the encephalic arteries; angiography (68) was also performed in one patient showing the occlusion of multiple small branches of the middle cerebral artery.

Anti-*Toxocara* antibodies in CSF were assessed in 24 cases (nineteen by ELISA, six by ELISA and WB, and two by WB). Out of the 19 patients assessed by CSF ELISA, 15 tested positive of whom three were also confirmed by positive WB; in two additional patients, the presence of anti-*Toxocara* antibodies was detected only by WB. In 10 patients, of whom 4 were vasculitis (62, 63, 67, 68), in which the diagnosis of neurotoxocariosis was based only on the presence of a positive serum ELISA seropositivity [CSF ELISA was negative in 5 (39, 47, 48, 59, 63) and not performed in the other 5 (53, 56, 62, 67, 68)], not confirmed by biopsy or WB (Table 1; Table S1 in Supplementary Material).

A mild–moderate eosinophilia was reported in 28 cases (84.9%); CSF analysis was performed in 24 patients of whom 17 had CSF pleocytosis (14 with eosinophilic pleocytosis). IgE levels were reported only in five patients and were elevated in four (Table S1 in Supplementary Material).

The diagnosis of encephalic/meningeal neurotoxocariasis was confirmed by biopsy in six cases (41, 42, 44, 50, 58, 60). Histopathological examination generally showed a granulomatous inflammatory response containing large numbers of eosinophils and neutrophils in the parenchyma with perivascular lymphocytic infiltrate. *Toxocara* larvae were detected in only two cases. The absence of larvae can be explained by the fact that the larvae are not permanently trapped by the reaction of the host but rather can escape, migrate, and trigger other inflammatory foci (59, 69). Out of these six confirmed cases, two presented with anti-*Toxocara* antibodies in the CSF. CSF analysis was not performed in four cases.

Twenty-three patients were treated with anthelmintic medications; seventeen received albendazole while the others received thiabendazole, mebendazole, or diethylcarbamazine. Fifteen of these patients were treated with concomitant corticosteroids. Clinical improvement with complete recovery was observed in all except two patients who suffered a relapse. The only death occurred in a 46-year-old woman with meningoencephalitis (53). However, it should be noted that in this case, eosinophilia and pleocytosis were absent and the diagnosis was based only on a positive serum IgG ELISA, not confirmed by biopsy (53). Two cases spontaneously improved without treatment (47, 52). Post-treatment brain MRI generally showed improvement, whereas antibodies were monitored in a few patients with variable results.

## Discussion

Toxocariasis is highly prevalent; however, neurotoxocariasis is rarely taken into account as a differential diagnosis in clinical settings. The diagnosis of neurotoxocariasis is a challenge because there is no distinct clinical syndrome. Imaging studies reported to date are not specific and serology has low specificity and sensitivity. In many reports, the diagnosis was only presumptive. Definitive diagnosis is given by histological confirmation, which is rarely available. Our systematic review demonstrated an impressive variation in case definitions and diagnostic evaluations. MRI findings in this large population were all suggestive, but none of them can be considered specific to neurotoxocariosis, and most of these findings can be seen in many other infectious or inflammatory conditions.

About 40% patients were diagnosed on the basis of serum IgG antibody detection by ELISA only. A positive IgG ELISA in serum to confirm the diagnosis may be of minimal value because of seroprevalence of 90%, particularly in tropical areas, and the ELISA frequently cross-reacts with other nematode infections (18, 20). Several studies have found that the antibody load decreases as time passes and after treatment (70). Thus, a more reliable marker of disease could be given by the normalization of antibody titers after treatment. Unfortunately, this finding was reported in only few cases with a wide variety of follow-up times, leading to inconsistent results.

On the other hand, peripheral eosinophilia was present in 64% and CSF pleocytosis in 40%. Their absence thus does not exclude CNS *Toxocara* involvement, an important finding because clinicians tend to look for eosinophilia as a basis to suspect a parasitic infection. Increased serum IgE levels were almost always positive, but reported in only 41% of the cases.

A diagnosis of neurotoxocariasis is also supported by clinical and MRI improvement after specific anthelmintic treatment. In 90% or more of cases, the drug used was albendazole. However, there are no specific guidelines for the treatment of neurotoxocariasis. Patients were treated with anthelmintics and corticosteroids in different dosage and administration schedules. In some cases, the improvement may have resulted from corticosteroid treatment that represents the first-line drug for several autoimmune inflammatory conditions characterized by similar MRI findings and thus would not necessarily support the diagnosis of neurotoxocariasis. Moreover, spontaneous remission has also been reported.

It follows that for a significant proportion of reported neurotoxocariasis cases, other infectious, particularly, parasitic or inflammatory etiologies cannot be definitively ruled out. In particular, in the case of myelitis, which comprised nearly 70% of the identified cases, other causes of autoimmune myelopathies should be suspected. Autoimmune myelopathies are a heterogeneous group of immune-mediated spinal cord disorders encompassing myelopathies with an immune attack on the spinal cord (e.g., aquaporin-4-IgG seropositive neuromyelitis optica), myelopathies occurring with systemic autoimmune disorders, paraneoplastic autoimmune myelopathies, postinfectious autoimmune myelopathies, and myelopathies considered to be immune related (e.g., multiple sclerosis and sarcoidosis) (71). These conditions are not distinguishable simply on the basis of MRI findings and can often improve after corticosteroid treatment. It should be emphasized that while a complete screening for other infectious conditions is almost always reported, screening for autoimmunity was performed only in few cases. For example, aquaporin-4 antibody was tested in just two cases.

Likewise, concerning cerebral toxocariasis, MRI often reveals the presence of a granulomatous process leading to reversible single or multiple ring-enhancing lesions. However, ring-enhancing lesions are a common finding that can be related to different disease processes such as infective, neoplastic, and inflammatory conditions. In particular, single enhancing lesions (SEL) represent a frequent diagnostic dilemma in tropical countries where they are generally due to infectious diseases such as neurocysticercosis and tuberculosis. In *T. solium* endemic areas, the presence of an SEL and its resolution after cysticidal drug therapy are considered to be major definitive criteria for the diagnosis of neurocysticercosis (72). However, single cysticercus granulomas are frequently seronegative for antibodies to *T. solium*, and in this scenario, a diagnosis of neurotoxocariasis cannot be entirely ruled out.

In conclusion, due to the lack of widely accepted, standard diagnostic criteria, there is a great variability in case definitions, diagnostic procedures, and diagnostic certainty for neurotoxocariasis. As suggested by data from animal models, larvae often migrate to the brain and neurotoxocariasis is probably less rare than assumed; like many tropical infectious diseases, it is often neglected and rarely diagnosed. MRI findings include single or multiple subcortical, cortical, or white matter hyperintense lesions, best visualized on FLAIR and T2-weighted imaging, and usually isointense or hypointense on T1. These imaging findings are suggestive but not specific to neurotoxocariasis. The main clinical manifestations of neurotoxocariasis are myelitis (present in approximately two-thirds of all reported cases), encephalitis, and meningitis. Neurotoxocariasis should be included in the differential diagnosis of cases of meningeal, cerebral, or spinal cord disease of unknown origin.

## Author Contributions

All authors (SS, HG, and AN) participated in conception and organization of review, literature search, and all stages of writing from initial draft to final product.

## Conflict of Interest Statement

The authors declare that the research was conducted in the absence of any commercial or financial relationships that could be construed as a potential conflict of interest. The reviewer ZN and handling editor declared their shared affiliation.
