# Supplementary material for: Clinical and Magnetic Resonance Imaging Findings of Neurotoxocariasis
Source: Front Neurol. 2018 Feb 8;9:53. doi: 10.3389/fneur.2018.00053 (PMC5809457; doi:10.3389/fneur.2018.00053)
Supplement: Supplementary file 1 [file table_1.doc]

Supplementary Material

**Clinical and MRI findings of Neurotoxocariasis**

**Sanchez SS, Garcia HH*****, Nicoletti A.**

*** Correspondence:** Garcia HH: hgarcia1@jhu.edu

# Table 1: Clinical characteristics of the 104 cases of neurotoxocariasis.

# Legend: * Diagnosis confirmed by biopsy; / not reported; n.p. performed; + positive; - negative; eosinophilia: + mild (500-1500), ++ moderate (1500-5000), severe (>5000); Pleo Eos: presence of pleocytosis eosinophila in CSF (not specified); EIA: Enzyme Immunoassay; IF: Immunofluorescence.

| **Author** | **Age** | **Clinical**  **presentation** | **MRI** | **ELISA**  **Serum** | **ELISA CSF** | **WB**  **Serum** | **WB**  **CSF** | **IgE** | **Peripheral Eosinophilia** | **CSF**  **N° cell (Eo %)** | **Treatment** | **MRI**  ***Follow-up*** | **Clinical**  ***Follow-up*** |  |
| --- | --- | --- | --- | --- | --- | --- | --- | --- | --- | --- | --- | --- | --- | --- |
| **Myelitis** | | | | | | | | | | | | | | |
| Abir 2017  (Case 2) | 66/F | Myelitis | Spinal MRI: T2WI hyperintensity and T1WI isointensity in postero-lateral region of the spinal cord Th5-Th7 and from Th10 toTh12 with contrast enhancement. Cervical lesion on C6. | + | + | + | + | 257 | ++ | 6  (65%) | Albendazole  Glucorticoid | Improved | Improved |  |
| Hiramatsu 2015 | 60/M | Myelitis | Spinal MRI: T2WI swelling and hyperintensity of the spinal cord from Th10 to the lumbosacral region. T1WI focal nodular enhancement in the posterior segment of the lumbar spinal cord. | + | + | + | + | 876 | + | 8  (21.5%) | Albendazole  Glucorticoid | Improved | Improved |  |
| Park  2012  (Case 1) | 71/M | Myelitis* | Spinal MRI: T2WI hyperintensity and swelling in the cervical spinal cord at the level of C3-C6. T1WI showed focal nodular enhancement on posterior segment at C5 level. | + | / | / | / | + | + | 0 | Albendazole  Glucorticoid | / | Stable |  |
| Park  2012  (Case 2) | 55/M | Myelitis | Spinal MRI: hyperintensity and mild cord swelling at C4-C6 level on T2WI with a nodular enhancement confined  to posterior column at C5 level | + | / | / | / | + | - | 0 | Albendazole  Glucorticoid | Recovery | Recovery |  |
| Fukae 2012 | 42/M | Myelitis | Spinal MRI: T2WI long cord lesions with hyperintensities at Th4 to Th6. T1WI swelling of the cord, and post-contrast focal enhancement at the Th5 level | + | + | / | / | 1635 | + | 8  (12.5%) | Albendazole  Glucorticoid | Improved | Improved |  |
| [Jabbour](https://www.ncbi.nlm.nih.gov/pubmed/?term=Jabbour RA%5BAuthor%5D&cauthor=true&cauthor_uid=21862933)  2011 | 17 cases | Myelitis | Spinal MRI: swelling and enlargement of the spinal segment; 7 were located in the cervical and 8 in the thoracic spinal cord. Lesions were isointense in T1WI and hyperintense on T2 and FLAIR sequences. Focal nodular enhancement was seen after intravenous gadolinium injection. The enhancing area involve the posterior marginal areas of the cord. | +  17/17 | +  17/17 | +  17/17 | +  17/17 | / | +  4/17 | 2/17  . | 14 Albendazole  Glucorticoid  3  DEC  Albendazole  Glucorticoid | Recovery | 50% Recovery  50% Improved |  |
| Ota  2010 | 5/M | Myelitis | Spinal MRI: Intramedullary lesion at the Th4, with edema from Th2 to Th6 | + | / | / | / | + | + | / | Albendazole | Improved | Improved |  |
| Lee HI  2010 | 8 cases | Myelitis | Spinal MRI: All were single lesion with high signal intensity on T2WI and minimal or mild swelling. All lesions showed focal nodular enhancement, especially in posterior or posterolateral segment. Three lesions were located in the cervical spinal cord and the other five lesions were located in the thoracic spinal cord | + | 2 +  (6 n.p.) | / | / | / | 5/8 | 1/8  (7 n.p.) | Albendazole  Glucorticoid  3/8  Glucorticoid  5/8 | / | Partially Improved |  |
| Lee YJ  2010 | 31 cases | Myelitis | Spinal MRI: All lesions were hyperintense on T2WI ; 78% showed involvement of more than 2 segments; 81.3% had spinal cord swelling and 75.0% showed nodular or patchy contrast enhancement.  Thoracic cord (43.8%) was the most frequently observed lesion, followed by the cervical (37.5%), both the cervical and thoracic (15.6%) and the lumbar cord (3.1%) | 31/31 | 10/10 | 8/11 | / | 31/31 | 17/31  + | 8/31  Pleo. Eos  (5 n.p.) | Albendazole  (20/31)  Glucorticoid  (31/31) | Improved | Improved (better with ALB) |  |
| Umehara  2006  (Case 2) | 40/F | Myelitis | Spinal MRI: T1WI revealed swelling of the spinal cord from Th1 to Th7; T2WI revealed high signal intensity at the corresponding levels. Focal enhancement was noted. | + | + | / | / | 802 | - | 9 (10%) | Albendazole  Glucorticoid | Improved | Improved |  |
| Dauriac-Le Masson  2005 | 32/F | Meningo  myelitis | Spinal MRI: intramedullar T2WI hyperintensity from Th6 to Th11, with a contrast-enhancement at level Th9 | + | + | / | / |  | - | 25 (50%) | Mebendazole | Recovery | Recovery |  |
| Eberhardt  2004 | 39/M | Meningo  myelitis | Spinal MRI: T2 hyperintense  intramedullary signal formation between C2 and Th1. The spinal cord itself appeared swollen. Contrast  enhancement was found in the central aspect of this lesion, most pronounced in C5/6. Slight leptomeningeal enhancement at the pontomedullary  and cervical level. | - | + | + | + |  | + | 13  (40%) | Albendazole  Glucorticoid | Recovery | Recovery |  |
| Radman  2000 | 42/F | Myelitis | Spinal MRI: Normal | + | / | + | / |  | +++ | 0 | Albendazole  Glucorticoid | / | Improved |  |
| Goffette  2000 | 40/F | Myelitis | Spinal MRI: T1WI hyperintense foci at Th8-Th10 with slight contrast-enahancement ; T2-weighted hyperintense foci Th8-Th10 | + | + | / | / |  | + | 26 (40%) | Mebendazole  Glucorticoid | Improved | Recovery |  |
| Strupp  1999 | 49/M | Meningo-  myelitis | Spinal MRI: T2WI hyperintense lesion in the thoracic cord which remained without enhancement after gadolinium injection on T1-weighted images | + | + | / | / | 166 | - | 128 (33%) | Albendazole  Glucorticoid | Improved | Improved |  |
| Duprez  1996 | 58/M | Myelitis | Spinal MRI: T2WI high-signal area from C2 to C5. Contrast-enhanced T1WI of the cervical spine with swelling of the cord and spotty enhancing foci in the posterior segment at C2 and C3. | +  (EIA) | / | / | / |  | ++ | 25 (50%) | Mebendazole  Glucorticoid | Improved | Improved |  |
| Kumar  1994 | 23/F | Myelitis | Spinal MRI: T1WI revealed spinal cord enhancement with gadolinium at the level of Th2-Th3 on the right. T2WI were unsatisfactory | +  (EIA) | + | / | / |  | ++ | Pleo. Eos | Thiabendazole  Mebendazole  Glucorticoid | Recovery | Improved |  |
| **Cerebral Toxocariasis** | | | | | | | | | | | | | | |
| Abir  2017  (Case 1) | 45/M | Cerebral toxocariasis | Brain CT scan: bilateral hypodense capsulo-lenticular lesions with focal nodular contrast enhancement.  Brain MRI: Hyperintense lesions on T2WI | + | + | + | + |  | ++ | 35  (57%) | Albendazole  Glucorticoid | Improved | Improved |  |
| Caldera  2013 | 54/M | Encephalitis | Brain MRI: Normal  Spinal MRI: Normal | / | / | + | +  (PCR) |  | ++ | Pleo. Eos. | Albendazole  Glucorticoid | / | Recovery |  |
| Keller  2008 | 7//M | Eosinophilic meningitis | Brain MRI: normal | +  (EIA) | +  (EIA) | / | / |  | + | Pleo. Eos. | Albendazole  Glucorticoid | / | Recovery |  |
| Kinčeková 2008 | 4/M | Cerebral toxocariasis | Brain MRI: Multiple irregular infiltrates within the periventricular white matter and in the frontal and occipital cortex. The lesions were hypointense on T1WI and showed hyper-intense signal on T2 and FLAIR images, with a confluent pattern | + | - | / | / |  | ++ | - | Albendazole  Mebendazole | Improved | Improved |  |
| Scheid  2008 | 45/M | Cerebral toxocariasis | Brain MRI: Polycystic lesion in the posterior and paramedian portion of the left thalamus. On T1WI the lesions appeared isointense. Post-gadolinium sequences showed no enhancement. | + | + | + | + | 26.4KU/I | - | 0 | Albendazole  Glucorticoid | Stable  (No effect) | Stable  (No effect) |  |
| [Gorgulu](https://www.ncbi.nlm.nih.gov/pubmed/?term=Gorgulu A%5BAuthor%5D&cauthor=true&cauthor_uid=16314947)  2006 | 56/F | Cerebral Abscess after meningioma aportation* | Brain MRI: mass enhanced with a ring pattern and perilesional edema (abscess?) in the tumor cavity. | + | / | / | / |  | / | / | Metronidazole | / | Recovery |  |
| Kazek  2006 | 7/F | Cerebral toxocariasis * | Brain MRI: low-density area within the white matter and cortex in the right posterior parietal lobe. Infusion of intravenous paramagnetic contrast showed a rim-enhancing lesion | + | / | / | / |  | + | / | Mebendazole | / | Improved |  |
| Moreira-Silva  2004  (Case 1) | 5/F | Cerebral toxocariasis | Brain MRI: small irregular lesions in the posterior portion of the spine-bulbar transition and pedunculus cerebellaris, hyperintense in T2 and DP. There was no enhancement after intravenous contrast | + | + | / | / |  | + | 187 (57%) | Thiabendazole  Albendazole | / | Recovery at 6 months |  |
| Bachili  2004 | 11/F | Cerebral toxocariasis * | Brain CT scan: hypodense cystic lesion, right parietal, with a diameter of 2 cm with perifocal edema.  Brain MRI: T1WI showed a thick diffuse ring enhancement and perifocal edema | + | / | / | / | + | + | 0 | Albendazole | Improved | Improved |  |
| Hoffmeister  2003 | 55/F | Cerebral toxocariasis | Brain MRI: Multiple cortical and subcortical hyperintense lesions in T2WI in both hemispheres. | + | + | / | / | 19986 | +++ | 0 | Albendazole  Glucorticoid  (2 cycles) | Improved | Improved  Relapse after 3 months |  |
| Vidal  2003 | 2/M | Eosinophilic Meningoencephalitis | Brain MRI: single lesion subcortically, which had a hyperintense, cloudy, and irregular appearance in T2WI | + | + | / | / |  | - | 161 (23%) | Albendazole  Glucorticoid | / | Improved |  |
| Zachariah  1994 | 61/M | Cerebral toxocariasis | Brain CT: abnormal areas of low attenuation in the left parietal and right hemispheric subcortical regions. After intravenous administration of a contrast agent, enhancement of the left parietal lesion.  Brain MRI: T2WI punctuate areas of increased signal, in the centrum semiovale and occipital white matter bilateral, Normal TlWI | +  (EIA) | - | / | / |  | ++ | 0 | Not performed | / | Spontaneus Improved |  |
| Ruttinger  1991 | 29/F | Cerebral toxocariasis | Brain MRI: 25 cortical or subcortical lesions hyperintense and irregular  on DP and T2WI. All lesions showed intense contrast-enhancement in their centres | + | - | / | / |  | + | 19 | DEC | Improved | Improved |  |
| Helsen  2011 | 45/M | Encephalomyelitis | Spinal MRI: T2WI hyperintensity Th4 –Th10 without contrast enhancement  Brain MRI: multiple T2WI hyperintense lesions. The supratentorial lesions showed contrast enhancement | + | + | / | / |  | ++ | 25  (9%) | Mebendazole  Albendazole | Recovery | Improved |  |
| [Moiyadi](https://www.ncbi.nlm.nih.gov/pubmed/?term=Moiyadi A%5BAuthor%5D&cauthor=true&cauthor_uid=17899691)  2007 | 38/M | Intracranial and intraspinal abscesses * | Brain and Spinal MRI:  multiple lesions in bilateral frontoparietal deep white matter and cervico medullary  junction. The lesions were hypointense on T1WI and hyperintense on T2WI suggestive of multiple abscesses.The lesions on contrast administration showed ring enhancement with perilesional edema | / | / | / | / |  | +++ | / | Not performed | / | / |  |
| Marx  2007 | 2/F | Encephalomyelitis (ADEM) | Brain and spinal MRI: FLAIR imaging showed multiple hyperintensity areas in the cerebral hemispheric white matter. T2WI showed extensive hyperintensity areas in the cervical spinal cord, without mass effect. No gadolinium enhancement was observed. | + | + | / | / |  | + | 277 (11%) | Thiabendazole  Glucorticoid | Recovery | Recovery |  |
| Umehara 2006  (Case 1) | 37/F | Cerebral and spinal toxocariasis | Spinal MRI: High signal intensity in the entire spinal cord at T2WI  Brain MRI: high signal intensity area in the left parietal white matter. | + | + | / | / |  | + | 53  (1%) | Albendazole  Glucorticoid | Improved | Initially  Improved  ,worsened after 6 months |  |
| Sick  2014 | 27/M | Eosinophilic meningitis | Brain MRI: Normal | + | + | / | / |  | - | 700  (64%) | Not performed | / | Spontaneus recovery |  |
| Cusguen 2013 | 46/F | Meningoencephalitis | Brain MRI: Communicating Hydrocephalus | + | / | / | / |  | - | 0 | Albendazole  Glucorticoid | / | worsened-died |  |
| Tobin  2011 | 42/F | Meningoencephalitis | Brain MRI: multiple white matter and periventricular hyperintense lesions on FLAIR sequences. T1WI gadolinium-enhanced contrast showed punctate, high signal density lesions surrounded by areas of low-signal vasogenic edema | + | + | / | / |  | + | 1220  (52.3%) | Albendazole  Glucorticoid | / | Recovery |  |
| Singer  2011 | 44/F | Meningoencephalo  myelitis | Brain MRI: Normal  Spinal MRI: T2WI showed several hyperintense lesions in the thoracic cord sparing the medullary conus. T1WI without and with gadolinium showing diffuse leptomeningeal and intraparenchymal enhancement | + | + | + | + |  | - | 51  (10%) | Albendazole  Glucorticoid | / | Improved |  |
| Salvador  2010 | 5/M | Cerebral toxocariasis and  polyneuropathy | Brain MRI: Normal  Spinal MRI: Enhancement of the cauda equina nerve roots with gadolinium on lumbosacral MRI | + | / | / | / |  | ++ | Pleo. Eos. | Thiabendazole | / | Recovery |  |
| Maiga 2007 | 73/M | Cerebral toxocariasis | Brain MRI: hyper-intense T2WI lesion on the brain stem (medulla, and mesencephalon) with a discrete enhancement after gadolinium injection. | + | / | + | + |  | +++ | 20  (23%) | Albendazole | Improved | Improved |  |
| Mrissa  2005 | 45/M | Meingoencephalitis* | Brain CT scan: Bilateral capsulolenticular hypodense lesions with central nodule contrast enhancement  Brain MRI: Lesions appeared in hyerintense on T2WI. | + | + | / | / |  | ++ | 35  (no eos) | Albendazole  Glucorticoid | Recovery | Recovery |  |
| Xinou  2003 | 54/F | Cerebral toxocariasis | Brain MRI: subcortical lesions in the right frontal and occipital lobes, and both centra semiovale. Lesions were hypointense on T1WI and hyperintense on T2WI and FLAIR sequences with intense contrast enhancement. Meningeal enhancement was observed close to the occipital lesion | + | - | / | / |  | +++ | 5/6 | Albendazole  Glucorticoid | Improved | Improved |  |
| Komiyama  1995 | 21/F | Cerebral toxocariasis and optic neuritis* | Brain and spinal MRI: lesions located mainly in cortical or subcortical layers of cerebrum, the cerebellum and upper cervical cord. Swelling and a gadolinium enhanced lesion of the right optic nerve. Lesions appeared hyperintense on T2WI and with contrast enhancement | + | + | / | / |  | ++ | 330 (30%) | DEC  Glucorticoid | / | Improved |  |
| Choi  2013 | 46/M | Obstructive hydrocephalus | Brain MRI: hydrocephalus and leptomeningeal enhancement at the prepontine cistern, left cerebellopontine angle cistern and bilateral cerebral hemisphere. Non-enhancing mass posterior to cerebral aqueduct | + | + | / | / |  | + | 0 | Albendazole  Glucorticoid  Shunt | Improved | Improved |  |
| Feske  2015 | 38/F | Vasculitis  (Eosinophilic endocarditis) | Brain MRI: DWI showed multiple (at least eight) small hyperintense lesions scattered  throughout both cerebral hemispheres, in white  and gray matter; the largest lesion was located in the left centrum semiovale, with no evidence of associated  intracranial mass or hemorrhage. The lesions  were thought to reflect multiple small acute infarcts. | + | / | / | / |  | + | / | Albendazole  Mebendazole | / | Recovery |  |
| [Kwon](https://www.ncbi.nlm.nih.gov/pubmed/?term=Kwon HH%5BAuthor%5D&cauthor=true&cauthor_uid=26157596)  2015 | 39/M | Multiple ischemic lesions | Brain MRI: DWI multifocal small acute infarctions in the internal border zone of both the cerebral hemisphere and left cerebellar hemisphere | + | - | / | / |  | ++ | 0 | Albendazole  Glucorticoid | / | Improved |  |
| Fellrath  2014 | 71/M | Vasculitis | Brain MRI: subcortical ischemic lesions | + | + | + | + |  | ++ | Pleo. Eos. | Albendazole  Glucorticoid | / | Improved |  |
| Lompo  2012 | 49/M | Vasculitis | Brain MRI: ischemic lesion in the superficial territory of the right ACA.  Angio-MRI: numerous segmental, irregular stenoses of the encephalic arteries | + | + | + | + | + | + | 15 | Albendazole  Glucorticoid | worsened | worsened |  |
| Helbok  2007 | 75/F | Vasculitis | Brain MRI: hyperintense lesions in DWI in the cerebrum and cerebellum and multiple contrast enhancing cortical and cerebellar lesions | + | + | + | + |  | ++ | 0 | Albendazole  Glucorticoid | Improved | Improved |  |
| Dousset 2003 | 25/M | Vasculitis | Brain MRI: Multiple cortical and white matter lesions. A recent hemorragic lesion was observed in the right semi-oval center hypointense in FLAIR in T2 and in diffusion. Other ischemic lesions as evidenced by the lowering of the diffusion coefficient on the ADC mapping | + | / | / | / |  | + | / | / | / | / |  |
| Sommer  1994 | 48/F | Vasculitis | Brain MRI: several hyperintense subcortical lesions in the cerebrum and cerebellum.  Angiography: Occlusion of multiple small branches of the middle cerebral arthery | +  (IF) | / | / | / | 3470 | ++ | Normal count but with eosinophilia. | Thiabendazole | Worsened | Worsened |  |
